# EP4 Agonist L-902,688 Suppresses EndMT and Attenuates Right Ventricular Cardiac Fibrosis in Experimental Pulmonary Arterial Hypertension

**DOI:** 10.3390/ijms19030727

**Published:** 2018-03-03

**Authors:** Ying-Ju Lai, I-Chen Chen, Hsin-Hsien Li, Chung-Chi Huang

**Affiliations:** 1 Department of Respiratory Therapy, Chang-Gung University College of Medicine, Tao-Yuan 33353, Taiwan; hsinhsien@mail.cgu.edu.tw (H.-H.L.); cch4848@adm.cgmh.org.tw (C.-C.H.); 2 Cardiovascular Division, Chang Gung Memorial Hospital, Tao-Yuan 33353, Taiwan; 3 Department of Respiratory Care, Chang-Gung University of Science and Technology, Chia-Yi 61363, Taiwan; 4 Respiratory Therapy and Chest Medicine, Cathay General Hospital, Taipei 10630, Taiwan; chenyichen0707@gmail.com; 5 Division of Thoracic Medicine, Chang Gung Memorial Hospital, Tao-Yuan 33353, Taiwan

**Keywords:** right ventricular fibrosis, EP4 agonist, EndMT

## Abstract

Right ventricular (RV) hypertrophy is characterized by cardiac fibrosis due to endothelial–mesenchymal transition (EndMT) and increased collagen production in pulmonary arterial hypertension (PAH) patients, but the mechanisms for restoring RV function are unclear. Prostanoid agonists are effective vasodilators for PAH treatment that bind selective prostanoid receptors to modulate vascular dilation. The importance of prostanoid signaling in the RV is not clear. We investigated the effects of the EP4-specific agonist L-902,688 on cardiac fibrosis and TGF-β-induced EndMT. EP4-specific agonist treatment reduced right ventricle fibrosis in the monocrotaline (MCT)-induced PAH rat model. L-902,688 (1 µM) attenuated TGF-β-induced Twist and α-smooth muscle actin (α-SMA) expression, but these effects were reversed by AH23848 (an EP4 antagonist), highlighting the crucial role of EP4 in suppressing TGF-β-induced EndMT. These data indicate that the selective EP4 agonist L-902,688 attenuates RV fibrosis and suggest a potential approach to reducing RV fibrosis in patients with PAH.

## 1. Introduction

Right ventricular (RV) hypertrophy and failure are major causes of morbidity and mortality in patients with pulmonary arterial hypertension (PAH) [1,2]. RV hypertrophy is characterized by cardiac fibrosis due to myocardial apoptosis, endothelial dysfunction, and endothelial–mesenchymal transition (EndMT) as well as the accumulation of collagen production [3]. These pathways have been therapeutically targeted with phosphodiesterase type 5 inhibitors (sildenafil), endothelin antagonists, soluble guanylate cyclase (sGC) stimulator (riociguat), and prostanoid agonists [2,4,5]. In particular, prostacyclin and its analogs (iloprost, beraprost, and treprostinil) have been shown to elicit beneficial effects in patients with pulmonary arterial hypertension (PAH) [6].

Prostaglandin E_2_ (PGE_2_) is an essential lipid mediator that activates four different prostanoid E (EP) receptors, including EP_1_, EP_2_, EP_3_, and EP_4_. In the vascular system, PGE_2_ is synthesized from arachidonic acid by cyclooxygenase (COX) enzymes [7,8]. Prostanoid receptors (IP, EP2, EP4, and DP) couple with stimulatory G protein (Gs) to elevate intracellular cyclic adenosine monophosphate (cAMP) and promote vascular relaxation [9]. Meanwhile, EP1, EP3, FP, and TP couple with inhibitory G protein (Gi) to reduce intracellular cAMP or elevate Ca^2+^ levels [9].

The predominant mechanism of a prostacyclin agonist in smooth muscle cells is to bind IP, which prompts adenylyl cyclase (AC) via Gs to convert ATP to cAMP, thereby activating the protein kinase A (PKA) pathway and vascular relaxation [9,10,11]. Prostacyclin treatment significantly increases cardiac output [5,8,12], improves functional capacity, and has been associated with improved survival [13]. However, a weakness of prostacyclin agonists is their short half-life [14], and long-term prostacyclin therapy induces receptor desensitization [15].

There is evidence that IP expression is decreased in the lungs of PAH patients [16,17], whereas EP4 expression remains stable [17]. Both IP and EP4 couple to Gs, which activates vascular relaxation [6,9]. Iloprost mediates vasodilation via EP4 under conditions of low IP expression, which occurs in PAH [17]. Many clinical studies have reported positive effects from the application of EP4 agonist in patients with lung disease, such as anti-inflammatory, anti-thrombotic, vasoprotective, and muscle dilator effects [18,19,20,21,22]. In addition, the EP4-specific agonist L-902,688 has been shown to reduce the RV hypertrophy and pulmonary arterial remodeling in hypoxic -PAH mice and monocrotaline (MCT)-induced PAH rats [23]. Targeted EP4 agonists may be a novel therapy for the treatment of PAH. Moreover, the role of EP4 in mediating RV hypertrophy associated with PAH is not well understood. Here, we examined collagen and α-smooth muscle actin (α-SMA) expression in the fibrotic right ventricles of MCT-induced PAH rats with or without treatment with an EP4-specific agonist, L-902,688, as well as the effect of L-902,688 treatment on TGF-β-induced EndMT, which is characterized by a loss of endothelial cell markers (eNOS, E-cadherin, and CD146) and an increase in mesenchymal and EndMT markers (Twist and α-SMA).

## 2. Results

### 2.1. EP4-Specific Agonist Prevents TGF-β-Induced EndMT In Vitro

**Figure 1:**
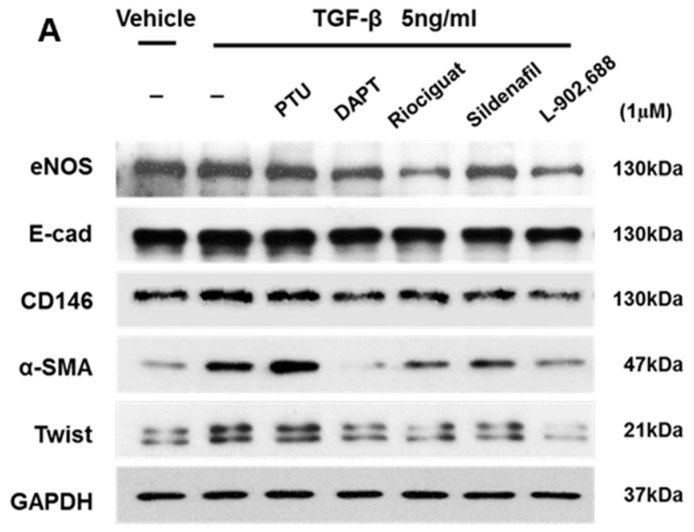

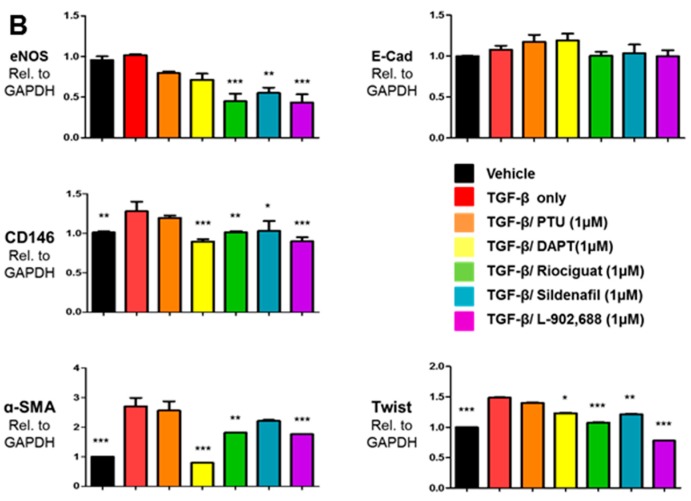
Effect of pulmonary arterial hypertension (PAH) drug candidates on endothelial cell markers and mesenchymal cell markers in cultured endothelial cells. (**A**) Representative immunoblot and (**B**) densitometric quantification of Western blot shows the expression levels of endothelial cell markers eNOS, E-cadherin (E-cad) and CD146, and mesenchymal cell markers Twist and α-smooth muscle actin (α-SMA) in whole cell extract. Human umbilical vein endothelial cells (HUVECs) were treated under the indicated conditions for 24 h. The relative expression level of each protein was quantified by densitometry and normalized to GAPDH. Each value represents the mean ± SE of three independent experiments. * *p* < 0.05, ** *p* < 0.01, and *** *p* < 0.001 versus the TGF-β1-only group (the red bar) by one-way ANOVA with Bonferroni’s post-test.

EndMT is involved in cardiac fibrosis, and is a multifaceted process whereby endothelial cells adopt a mesenchymal phenotype and overexpress mesenchymal cell markers such as α-SMA and Twist [24,25]. The endocardial layer may contribute to ventricular fibrosis via EndMT to cause RV hypertrophy in the MCT-PAH rat model. TGF-β signaling has been shown to play an important role in EndMT [3,25]. In fact, adding TGF-β to epithelial cells in culture is a convenient way to induce EndMT in various epithelial cells [25]. Therefore, we utilized cultured human umbilical vein endothelial cells (HUVECs) to test the suppressive effect of preclinical and clinical PAH drug candidates such as propylthiouracil (PTU) (Sigma, Saint Louis, MO, USA) [26], *N*-[*N*-(3,5-difluorophenacetyl)-l-alanyl]-*S*-phenylglycine *t*-butyl ester (DAPT, a γ-secretase inhibitor; Calbiochem, La Jolla, CA, USA) [27], riociguat (Cayman Chemical, Ann Arbor, MI, USA) [28], sildenafil (Cayman Chemical) [29], and L-902,688 (Cayman Chemical) at 1 µM against 5 ng/mL TGF-β-induced EndMT. Western blot analysis of endothelial cell marker (eNOS, E-cadherin, and CD146) and mesenchymal and EndMT marker (Twist and α-SMA) on TGF-β1-induced EndMT (Figure 1A) expression showed that α-SMA and Twist expression levels were strongly decreased by treatment with 1 µM L-902,688 (Figure 1B). Taken together, these data suggest that the inhibitory effect of the EP4 agonist L-902,688 on RV fibrosis might be mediated by suppression of α-SMA and Twist, the EndMT markers.

### 2.2. The Role of EP4 in L-902,688-Induced Suppression of TGF-β-Induced EndMT

**Figure 2:**
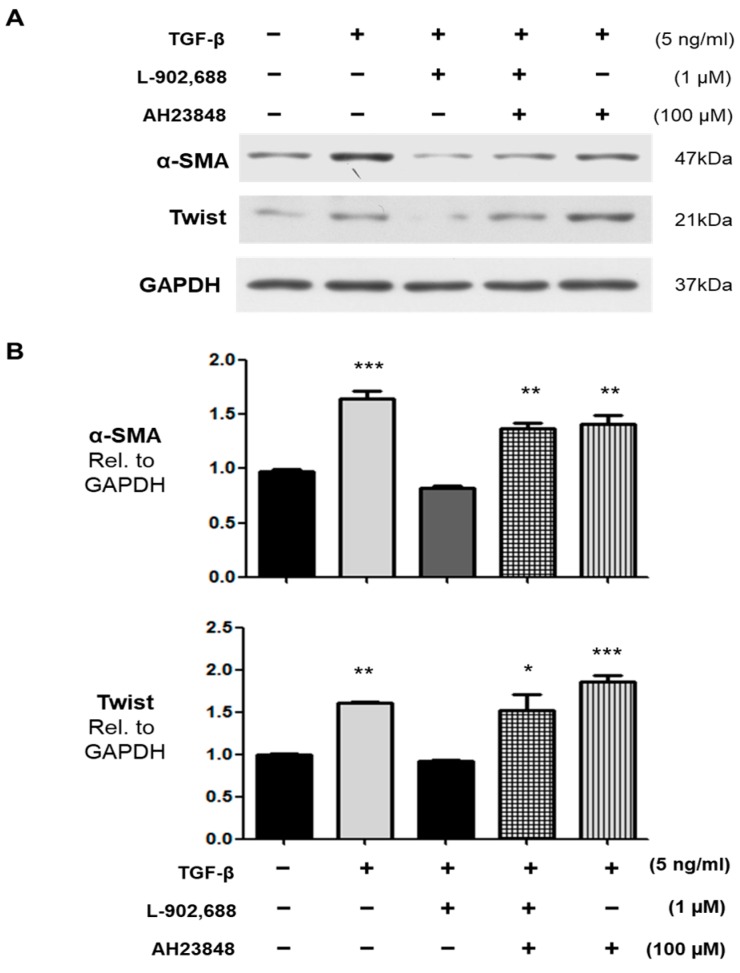
The EP4 inhibitor AH23848 blocks the L-902,688-induced suppression of TGF-β-induced EndMT in HUVEC. TGF-β-induced expression of the EndMT markers α-SMA and Twist in endothelial cells was suppressed by L-902,688. The endothelial cells were stimulated for 30 min with 100 µM AH23848 with or without L-902,688 or TGF-β for 24 h. Data are the mean ± SE of three different experiments. * *p* < 0.05, ** *p* <0.01, and *** *p* < 0.001 versus untreated group. (**A**) Representative immunoblots showing that the suppression of TGF-β-induced expression of α-SMA and Twist by 1 µM L-902,688 was reversed with an EP4 inhibitor (AH23848) at 100 µM. (**B**) Densitometry quantification of α-SMA and Twist expression following EP4 inhibitor treatment in endothelial cells.

**Figure 3:**
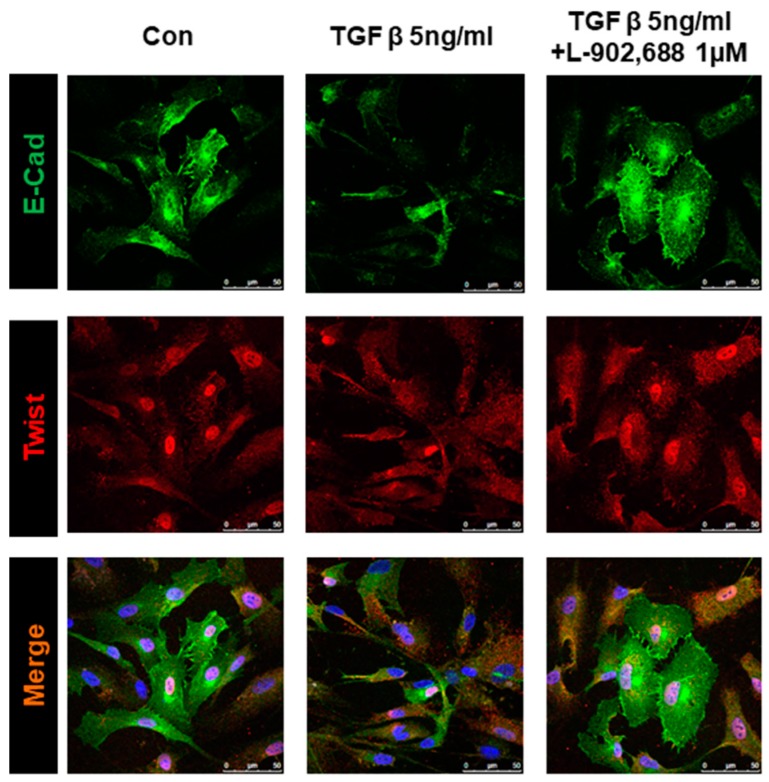
Effect of L-902,688 on TGF-β-induced Twist expression in HUVEC. After 24 h of serum deprivation, endothelial cells were treated under the indicated conditions for 24 h. Immunocytochemistry shows the localization of Twist (red) and E-cadherin (E-cad) (green) expression (nuclear staining with DAPI in blue; scale bar: 50 µm). The images are a representative of three independent experiments.

The effects of L-902,688—an EP4 agonist—are mainly mediated by EP4 receptors. TGF-β signaling has been shown to play an important role in EndMT [3,25]. To determine the contribution of EP4 to L-902,688-induced suppression of TGF-β-induced EndMT, we treated cultured endothelial cells with the EP4 antagonist AH23848 with or without L-902,688, thereby investigating whether L-902,688 attenuates TGF-β1-induced EndMT by activating the EP4 signaling pathway. Pre-incubation with the EP4 inhibitor AH23848 blocked L-902,688-induced EP4 signaling, as exposure to AH23848 (100 μM) with or without TGF-β (5 ng/mL) reversed the L-902,688-induced inhibition of α-SMA and Twist expression compared with HUVEC not treated by L-902,688 and TGF-β (Figure 2A,B), thus reflecting a crucial role for EP4 and the TGF-β1-induced EndMT pathway in the effect of L-902,688. Immunocytochemistry showed that L-902,688 inhibited the expression of Twist, the TGF-β1-induced EndMT marker, in HUVEC (Figure 3). These results indicate that L-902,688 attenuated TGF-β1-induced EndMT by activating EP4 signaling.

### 2.3. Effect of L-902,688 on Right Ventricular Changes in Experimental PAH

**Figure 4:**
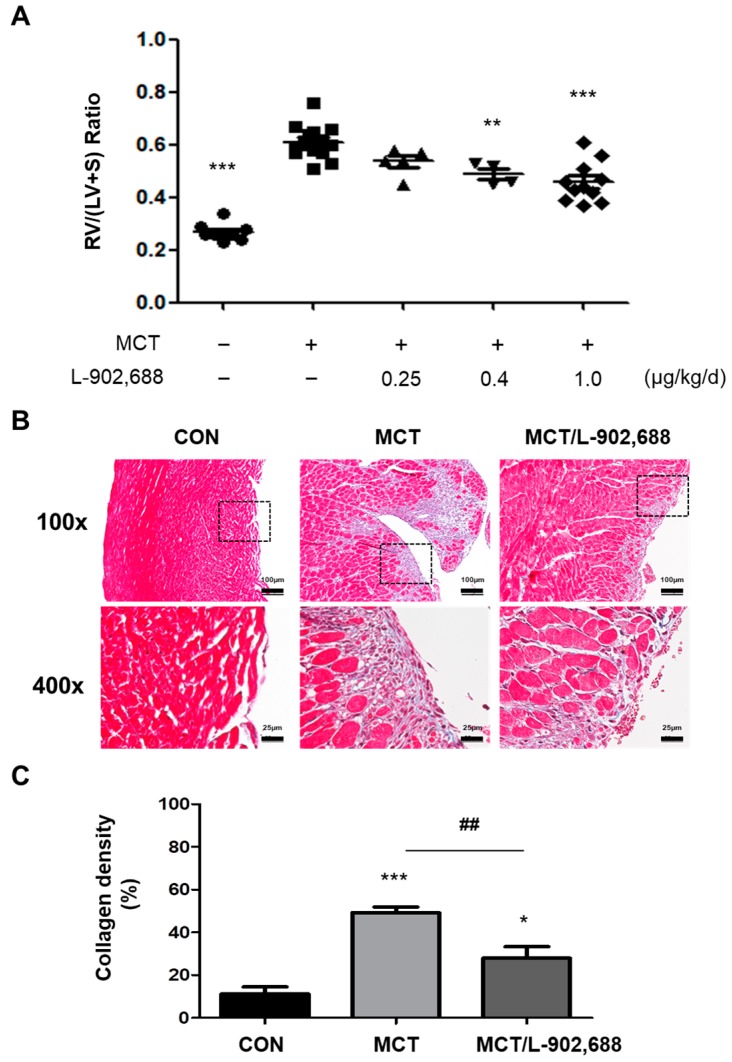
Histological analysis of trichrome-stained right ventricular (RV) tissue. (**A**) Administration of L-902,688 from the 8th to the 28th day after monocrotaline (MCT) injection decreased the RV/(LV + S) weight ratio in MCT-induced PAH rats. (CON *n* = 10, MCT *n* = 15, MCT/L-902,688 0.25 µg/kg/day *n* = 5, MCT/L-902,688 0.4 µg/kg/day *n* = 4, MCT/L-902,688 1.0 µg/kg/day *n* = 11). ** *p* < 0.01 and *** *p* < 0.001 versus MCT group. (**B**) The histological analysis of trichrome-stained RV tissue demonstrated greater collagen (purple) deposition in the endocardium of MCT rats at week 4 than in that of control (CON) rats. EP4 agonist 1 µg/kg/day (MCT/L-902,688) treatment significantly reduced collagen deposition. Scale bars = 100 and 25 μm. (**C**) Quantitative analysis of collagen deposition by a color deconvolution method demonstrated a significant reduction in the percentage of fibrosis among the total tissue area. Bars represent the mean ± SE (*n* = 5 per group); * *p* < 0.05 and *** *p* < 0.001 versus CON; ## *p* < 0.01 versus the MCT group.

EP4-specific agonist L-902,688 (1 µg/kg/day) in the MCT-induced PAH rat was shown to reduce the RV hypertrophy [23]. The next experiment was designed to evaluate whether the inhibitory effect of L-902,688 on EndMT in vitro could attenuate RV changes in vivo. The efficacy of L-902,688 (0.25, 0.4 and 1 µg/kg/day) and changes in RV structure was evaluated in an MCT-induced PAH rat model. As expected, rats challenged with MCT developed RV hypertrophy—as indicated by an increased weight of the right ventricle (RV) divided by weight of the left ventricle (LV) plus septum (S) (RV/(LV + S) weight ratio)—on the 28th day after MCT injection compared with controls (Figure 4A). Administration of L-902,688 from the 8th to the 28th day after MCT injection decreased the RV/(LV + S) weight ratio (Figure 4A) in MCT-induced PAH rats. The histological analysis of trichrome-stained RV tissue demonstrated a striking difference in the amount of collagen deposition between the L-902,688 and MCT groups (Figure 4B). Quantification of the collagen (shown in blue) area compared to the total tissue area demonstrated a significant difference between the two groups (Figure 4C).

### 2.4. EP4-Specific Agonist Prevents Cardiac Fibrosis and Inhibits α-SMA Expression in Hypertrophic RV Tissue

**Figure 5:**
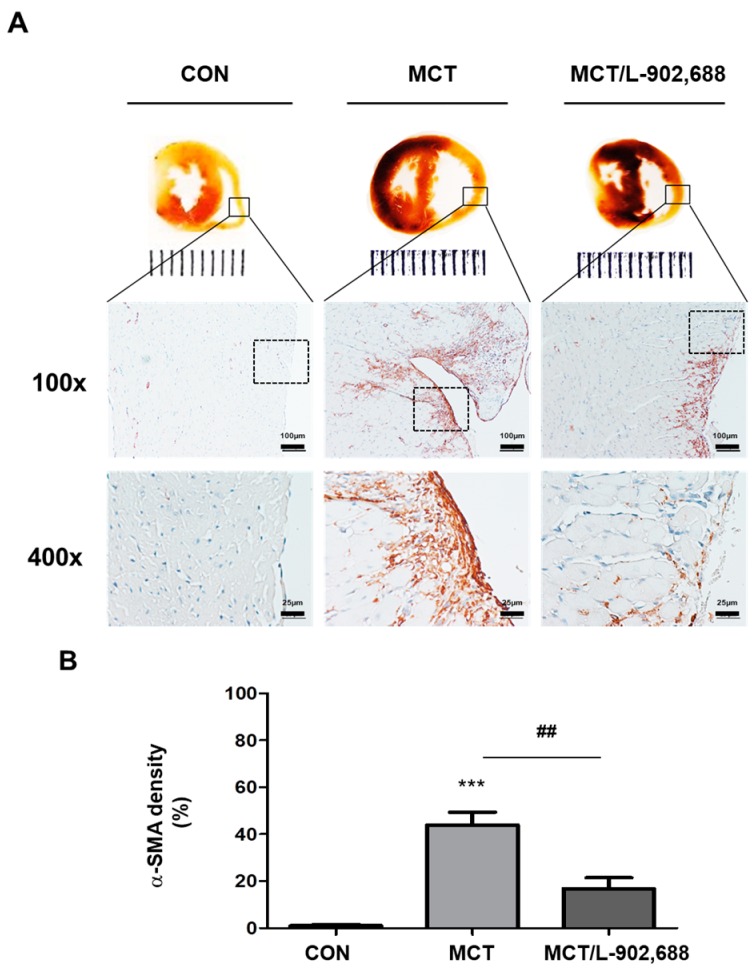
Histological analysis of the mesenchymal cell marker α-SMA in right ventricular (RV) tissue. (**A**) Cardiac morphology in cardiac cross-sections and histological analysis of α-SMA expression in RV tissue demonstrated that endocardial α-SMA (brown) expression was significantly higher in MCT rats at Week 4 than in control (CON) rats. However, 3 weeks of EP4 agonist 1 µg/kg/day (MCT/L-902,688) treatment significantly reduced α-SMA (brown) expression. In the top panel, scale bars = 100 μm; in the bottom panel, scale bars = 25 μm. (**B**) Quantitative analysis of α-SMA (brown) expression by a color deconvolution method demonstrated a significant reduction in the percentage of α-SMA (brown) among the total tissue area. Bars represent the mean ± SE (*n* = 5 per group); *** *p* < 0.001 versus CON; ## *p* < 0.01 versus the MCT group.

Cardiac fibrosis is associated with a loss of microvasculature and with the disruption of myocardial structures, and is caused by extreme deposition of extracellular matrix, which is mediated by mesenchymal cells or fibroblasts [3]. Furthermore, there is evidence that IP expression is decreased in the lungs of PAH patients [14,15], whereas EP4 expression remains stable [17]. We hypothesized that a targeted EP4 agonist could be a novel therapeutic for the treatment of PAH. Critical stages in the progression of cardiac fibrosis include the transformation of fibroblasts into myofibroblasts and EndMT [24,30]. Myofibroblasts are smooth muscle-like fibroblasts that synthesize collagen and express α-SMA protein [31]. TGF-β signaling has been shown to play an important role in EndMT [25]. Furthermore, EndMT in cardiac fibrosis is a multifaceted process whereby endothelial cells lose their specific markers (e.g., eNOS, E-cadherin, and CD146) and adopt a mesenchymal or myofibroblastic phenotype, overexpressing mesenchymal cell markers such as α-SMA and collagen [24]. To examine whether the EP4 agonist L-902,688 affects cardiac fibrosis associated with PAH, MCT rats were treated with L-902,688 for 21 days, and α-SMA was detected by immunohistochemical staining. Immunohistochemistry showed that MCT rats exhibited higher expression levels of α-SMA in the endocardium of the right ventricle than control (CON) rats (Figure 5A). Notably, there was a gradual decrease in α-SMA expression in the endocardium of the right ventricle of L-902,688-treated MCT rats (Figure 5A). Quantification of the α-SMA expression (shown in brown) area compared to the total tissue area demonstrated a significant difference between the two groups (Figure 5B), possibly reflecting that L-902,688 may prevent cardiac fibrosis in the right ventricle. Thus, EP4 signaling plays a critical role in regulating cardiac fibrosis.

## 3. Discussion

The novel finding of this study is that EP4-specific agonist therapy reduced right ventricular (RV) fibrosis, as evaluated by Masson’s trichrome staining, and inhibited α-smooth muscle actin (α-SMA) expression in hypertrophic RV tissue. In addition, EP4-specific agonist therapy prevents TGF-β1-induced endothelial–mesenchymal transition (EndMT). Here, we compared preclinical and clinical PAH drug candidates, including DAPT (γ-secretase inhibitor) [27], riociguat [28], sildenafil [29], and L-902,688, and showed that L-902,688 prevented TGF-β1-induced EndMT, which is characterized by an increase in mesenchymal and EndMT markers such as Twist and α-SMA. These data indicate that the selective EP4 agonist L-902,688 attenuates RV cardiac fibrosis by suppressing EndMT and that increasing EP4 signaling in the RV might be an approach to reducing RV fibrosis and improving RV function in patients with pulmonary hypertension.

RV hypertrophy and dysfunction cause morbidity and mortality in patients with pulmonary arterial hypertension (PAH). Natriuretic peptides, including atrial natriuretic peptide (ANP) and brain natriuretic peptide (BNP), are recognized as useful biomarkers for ventricular dysfunction, and are increasingly being employed as biomarkers in PAH and RV dysfunction [32]. Prostacyclin and its agonist (iloprost) are first-line treatments for patients with PAH, significantly decreasing ANP [33] and significantly increasing cardiac output [5,8,12], which is associated with improved survival [13]. The major mechanism of prostacyclin agonists in smooth muscle cells is to bind IP, which activates adenylyl cyclase (AC) via Gs to convert ATP to cAMP, leading to activation of the protein kinase A (PKA) pathway and vascular relaxation [9,10]. However, the weakness of prostacyclin agonists is their short half-life [14], and long-term prostacyclin therapy induces receptor desensitization [15]. Furthermore, there is evidence that IP expression is disrupted in the lungs of PAH patients [16,17], whereas EP4 expression remains stable [17]. In the transgenic mouse model, IP receptor deficiency leads to the development of significant cardiac fibrosis [34,35]. Both IP and EP4 couple to Gs, which activates vascular relaxation [6,9]. Iloprost mediates vasodilation via EP4 under conditions of low IP expression associated with PAH [17]. Therefore, a targeted EP4 agonist may be a novel therapeutic for the treatment of RV hypertrophy and dysfunction in patients with PAH.

Interestingly, numerous EP4 agonists have been reported to be involved in cardiac disease and fibrosis in vivo. EP4 agonists have been shown to exhibit antifibrotic effects via protein kinase A (PKA) activation and prevent the progression of left ventricular systolic dysfunction in hypertrophied hearts [36]. In addition, EP4 agonist treatment in mice subjected to myocardial infarction and ischemia/reperfusion reduced the myocardial infarct size and preserved cardiac dysfunction [37,38]. EP4 agonist treatment also prevented kidney fibrosis in a mouse model of chronic kidney disease [39]. These studies indicate that EP4 agonists have antifibrotic and organ-protective effects. Therefore, we expected that the EP4-specific agonist L-902,688 would also show antifibrotic ability when administered during RV hypertrophy with preserved PAH. Adult male Sprague-Dawley rats were randomized for treatment 28 days after a single injection of crotaline to induce PAH and RV hypertrophy. After 1 week, we administered L-902,688 until the 4th week, which reduced the expression level of the EndMT signaling marker α-SMA and collagen production in MCT-induced RV hypertrophy. Therefore, we demonstrated by Masson’s trichrome staining that an EP4-specific agonist—L-902,688—significantly reduced RV fibrosis in the MCT rat model of severe RV failure.

PAH is a complex progressive disease with three major therapeutic targets: the endothelin, nitric oxide, and prostacyclin (PGI_2_) pathways [4,7]. Current strategies for PAH treatments recommend combining multiple pathways in parallel. Double combination therapy in PAH is now the standard of care for patients [40] and is becoming common in clinical practice to delay progression of the disease [41]. This can target the endothelin pathway by endothelin receptor antagonists (ERAs) such as bosentan, ambrisentan, or macitentan, and simultaneously combining with treatments for the nitric oxide pathway through PDE-5 inhibitors, including sildenafil and tadalafil, and more recently, the soluble guanylate cyclase stimulator riociguat [42]. Treatments targeting the prostacyclin pathway include epoprostenol, iloprost, treprostinil, and beraprost [43,44]. However, current combination therapies do not specifically target RV function. RV cardiac fibrosis is due to EndMT and overexpression of mesenchymal cell markers such as α-SMA and Twist [24,30]. To the best of our knowledge, these findings are the first to identify and compare with preclinical and clinical PAH drug candidates such as PTU [26], DATP [27], riociguat [28], and sildenafil [29], regarding the preventative effect of TGF-β1-induced EndMT in vitro relative to combination therapies for PAH. Western blot analysis of the expression of endothelial cell markers eNOS, E-cad, and CD146, and mesenchymal markers Twist and α-SMA on TGF-β1-induced EndMT showed that both of α -SMA and Twist expression was strongly decreased by 1 µM L-902,688, DAPT, sildenafil, and riociguat treatment. The cardioprotective and antifibrotic effects of sildenafil and riociguat have been confirmed in previous studies [45,46,47]. In our study, we demonstrated that L-902,688 significantly decreased Twist and α-SMA protein expression in TGF-β1-induced EndMT.

Our results reveal that the EP4 agonist L-902,688 inhibits mesenchymal marker (Twist and α-SMA) expression and partially reverses established RV cardiac fibrosis. Thus, EP4 signaling may be beneficial for reversing cardiac fibrosis independent of a reduction in RV pressure overload.

## 4. Materials and Methods

### 4.1. Experimental Design

Adult male Sprague-Dawley rats were randomized for treatment 28 days after a single subcutaneous injection of saline or 60 mg/kg crotaline (Sigma, Saint Louis, MO, USA) to induce PAH and RV hypertrophy. After 1 week, the experimental rats received once-daily intraperitoneal injections of L-902,688 (Cayman Chemical, Ann Arbor, MI, USA) at a dosage of 0.25, 0.4 or 1 µg/kg/day or saline as the vehicle control. Rats were examined after 3 weeks of treatment (on day 28). L-902,688 (EP4 agonist) was used to target the EndMT signaling marker α-SMA in MCT-induced rats with right ventricle hypertrophy, and the dose was calculated based on published studies [48]. All institutional and national guidelines for the care and use of laboratory animals were followed, and the protocol was approved by the Chang Gung University institutional committees IACUC (permit number: CGU15-103, 16 January 2016 approved). Animal housing and maintenance were provided by Chang Gung University, and all animals were fed a standard chow diet with free access to water.

### 4.2. Modified Masson’s Trichrome Staining

Rat hearts were collected and fixed with buffered 10% paraformaldehyde (PFA)*.* Before immunostaining, the slides were deparaffinized (xylene), washed with alcohol (100%, 95%, 75%, 50%, and 35%), and then rehydrated in deionized water. Sections for Modified Masson’s trichrome staining were processed according to the manufacturer’s instructions (ScyTek, Logan, UT, USA).

### 4.3. Immunohistochemistry

In a separate set of immunohistochemistry (IHC) experiments using the Dako LSAB peroxidase kit (Dako, Santa Clara, CA, USA), rat heart sections were incubated for 10 min in −20 °C methanol and 3% hydrogen peroxide. The slides were permeabilized and blocked for 30 min in 1% bovine serum albumin (BSA) (Sigma, Kanagawa Prefecture, Japan) and then incubated for 30 min with primary antibodies against α-SMA (dilution 1:1000, Thermo, Waltham, MA, USA) in antibody diluent (Dako). Slides were rinsed with 1 × PBS and incubated for 30 min with a streptavidin-biotin system (Dako) to detect the signals; brown color development was evaluated following incubation with diaminobenzidine (DAB) substrate-chromogen for 1 min (EnVision/HRP, Dako). Finally, after rinsing with deionized water, the slides were counterstained with hematoxylin, dehydrated, mounted, and coverslipped. Staining of α-SMA was used to indicate RV cardiac fibrosis.

### 4.4. Human Umbilical Vein Endothelial Cells (HUVECs) Culture

HUVECs were cultivated in 0.1% gelatin-coated 100-mm dishes with endothelial cell growth medium (Lonza, Basel, Switzerland) and used between passages 5 and 7. Cells were starved in endothelial cell basal medium (0.01% FBS) (Lonza) for 24 h before the experiment.

### 4.5. Western Blot Analysis

HUVECs were exposed to the EP4 antagonist (AH23848; 100 μM, Sigma, Kanagawa Prefecture, Japan) with or without L-902,688 (1 µM) or TGF-β (5 ng/mL) for 24 h. Western blotting was performed using anti-eNOS (BD), anti-E-cadherin (E-cad) (Cell Signaling, Danvers, MA, USA), anti-CD146 (Abcam, Cambridge, UK), anti-α-SMA (Thermo, Waltham, MA, USA), and anti-Twist (Novus, Frauenfeld, Switzerkand) primary antibodies. Peroxidase-conjugated anti-mouse IgG or anti-rabbit IgG (Cell Signaling) were administered as secondary antibodies. Blots were visualized using the enhanced chemiluminescence detection system (Amersham, UK). Samples were normalized to GAPDH (Santa Cruz, CA, USA) and quantified by densitometry.

### 4.6. Immunocytochemical Analysis

Immunocytochemical analysis of HUVECs was performed with primary antibodies against E-cadherin (E-cad) (Cell Signaling, Danvers, MA, USA) and Twist (Novus, Frauenfeld, Switzerkand). At the end of the experiments, cells were fixed with cold methanol, blocked with 1% goat serum/1% BSA in PBS for 30 min, and then incubated with primary antibodies for 1 h. Subsequently, cells were incubated with Alexa-488-conjugated (green) and Cy3-conjugated (red) secondary antibodies to label E-cadherin and Twist, respectively. Nuclei were visualized by DAPI staining (Gibco, Invitrogen, Carlsbad, CA, USA). Fluorescence was observed with a confocal microscope (Confocal TCS SP8XL; Leica, Wetzlar, Germany) at the Microscope Core Laboratory of Chang Gung Memorial Hospital.

### 4.7. Statistical Analysis

The data are presented as the mean and standard error (SE). One-way ANOVA with post hoc Bonferroni’s test was used to compare the data of multiple groups. *p* ≤ 0.05 was considered statistically significant.
